# Biochar application to rice with ^15^N-labelled fertilizers, enhanced leaf nitrogen concentration and assimilation by improving morpho-physiological traits and soil quality

**DOI:** 10.1016/j.sjbs.2021.03.003

**Published:** 2021-03-14

**Authors:** Saif Ullah, Quan Zhao, Ke Wu, Izhar Ali, He Liang, Anas Iqbal, Shanqing Wei, Fangwei Cheng, Shakeel Ahmad, Ligeng Jiang, Syeda Wajeeha Gillani, Amanullah, Shazma Anwar, Zaid Khan

**Affiliations:** a Key Laboratory of Crop Cultivation and Farming System, Guangxi University, Nanning 530004, China; b State Key Laboratory for Conservation and Utilization of Subtropical Agro-bioresources, Guangxi University, Nanning 530004, China; c Department of Agronomy, The University of Agriculture Peshawar, Peshawar 25130, Pakistan

**Keywords:** Rice, Biochar, Nitrogen concentration, Root morphology, Photosynthetic pigments

## Abstract

Leaf nitrogen (N) concentration plays an important role in biochemical and physiological functions, and N availability directly influences rice yield. However, excessive N fertilization is considered to be a root cause of environmental issues and low nitrogen use efficiency. Therefore, the selection of appropriate nutrient management practices and organic amendments is key to maximizing nitrogen uptake and maintaining high and sustainable rice production. Here, we evaluated the effects of different ^15^N-labelled nitrogen sources (urea, ammonium nitrate, and ammonium sulfate at 315 kg ha^−1^) with or without biochar (30 t ha^−1^) on paddy soil properties, root growth, leaf gas exchange, N metabolism enzymes, and N uptake in the early and late seasons of 2019. We found significant differences among N fertilizer sources applied with or without biochar (*P* < 0.05). Across the seasons, the combination of biochar with N fertilizers significantly increased soil organic carbon by 51.21% and nitrogen availability by 27.51% compared with N fertilizers alone. Correlation analysis showed that rice root morphological traits were strongly related to soil chemical properties, and higher root growth was measured in the biochar treatments. Similarly, net leaf photosynthetic rate averaged 9.34% higher, chlorophyll (Chl) a concentration 12.91% higher, and Chl b concentration 10.05% higher in the biochar treatments than in the biochar-free treatments across the seasons. Notably, leaf ^15^N concentration was 23.19% higher in the biochar treatments in both seasons. These results illustrated higher activities of N metabolism enzymes such as NR, GS, and GOGAT by an average 23.44%, 11.26% and 18.16% in the biochar treatments across the seasons, respectively. The addition of biochar with synthetic N fertilizers is an ecological nutrient management strategy that can increase N uptake and assimilation by ameliorating soil properties and improving the morpho-physiological factors of rice.

## Graphical abstract

**Figure:**
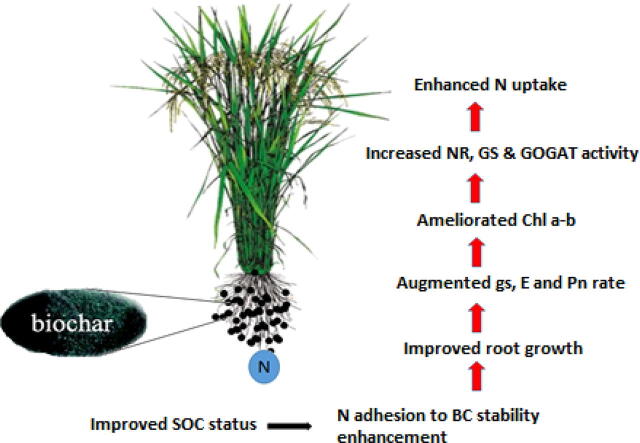

## 1 Introduction

Rice (*Oryza sativa* L.) is among the most globally important cereal crops and provides essential nourishment for more than half the world’s population (Carrijo et al., 2017, Chauhan et al., 2017). China is a main rice producing nation in the world, contributing 28.7% of the worldwide rice production, as about 65% of Chinese people rely on rice and the means crop as source of income for the vast majority of the farmers (Li et al., 2009). The increasing population has created a demand for 20% more rice production by 2030 to meet domestic need (FAOSTAT, 2019). Grain filling is a significant determinant of grain yield and rice productivity; it is characterized by the duration and rate of grain filling and the uptake of nutrients (Chang and Zhu, 2017, Huang et al., 2017a). Plant leaves perform two vital roles during grain filling in cereal crops: they are essential photosynthetic organs necessary for the generation of dry matter, and they are a key source of nutrients for grain filling (Sanchez-Bragado et al., 2016). Inadequate accumulation of nitrogen (N) in plant leaves and rapid N translocation from leaves accelerate leaf senescence and diminish leaf photosynthetic efficiency, resulting in less assimilation to the grain (Yang & Zhang, 2010). Chlorophyll *a* and b are the core photosynthetic pigments of the chloroplasts and have critical roles in the assimilation and manipulation of light energy, thereby affecting photosynthetic productivity (Zhang et al., 2009). Augmenting chlorophyll in crops may be an effective means of increasing biomass production and grain yield (Wang et al., 2008). Fewer studies have been performed to understand the role of nitrogen regulation and metabolism enzymes (Forde & Lea, 2007), particularly enzymes such as nitrate reductase (NR), glutamine synthetase (GS), and glutamine oxoglutarate amoinotransferase (GOGAT) that play key roles in primary N assimilation (Krapp et al., 2011). These nitrogen-containing enzyme complexes regulate N uptake, absorption, and metabolism within the plant in response to external N accessibility. The fundamental enzymes of nitrogen assimilation and the end products of N metabolism are the most important biochemical factors that influence nitrogen use efficiency (NUE) (Xu et al., 2012). This appears to be a useful cycle. Thus, monitoring the activity of these enzymes will provide insight into the relationship between N metabolism enzymes and N uptake.

Biochar is a carbonaceous product derived from the incomplete combustion of various organic materials such as crop straw; it is currently attracting increased attention worldwide for its role in promoting the sustainable development of resources, the environment, and agriculture. Several studies have reported the impact of biochar addition on plant growth and development. In general, biochar application increases soil N accessibility and retention, reduces soil bulk density, enhances water holding capacity, increases pH and cation exchange capacity, encourages useful microorganisms, and restricts the bioavailability of heavy metals, all of which are ultimately associated with increased plant photosynthesis (Chen et al., 2019, Lehmann et al., 2011). Moreover, biochar addition induces changes in soil properties that can influence plant performance by altering root growth and associated characteristics. Studies show that biochar addition improved plant growth yield, improved water quality, decreased nutrients leaching, diminished soil acidity, enhanced water maintenance, and lessened irrigation and fertilizer necessities. The plant take-up of key nutrients ultimately increase plant growth and yield because of biochar amendment, especially when within the presence of added nutrients (Woods et al., 2006, Taghizadeh-Toosi et al., 2012). However, little work has been conducted on the effect of biochar on fertilizer N-use effectiveness.

Roots are the primary source of contact between biochar particles and plants; yet, studies conducted mainly focused on above ground biomass and yields in response of plant to soil applied with biochar. Plant response to biochar might be through a direct linkage between biochar particles and roots. Fine roots, root hairs or mycorrhizal fungal hyphae may take up nutrients, toxins or water from surfaces or from internal biochar pores. Indirect biochar –root interactions could develop from: impacts on soil biogeochemistry (pH, nutrient availability, aeration or water holding capacity (WHC); Jones et al. (2012), activity of their surrounding community and arrangement (Rondon et al., 2007), and delivery or sorption of chemical signals influencing root development (Spokas et al., 2009). These direct or indirect signaling between biochar particles and root could start a scope of reactions in root systems and influence plant growth. A growing body of literature suggests that biochar addition invigorates root growth and improves root morphological traits, including root biomass (RB), volume, surface area, density, and length, enabling the plant to acquire more nutrients and water, thereby stimulating plant growth (Bruun et al., 2014, Changxun et al., 2016). Biochar application with mineral fertilizer increments photosynthetic rate (Pn), stomatal conductivity, cellular CO_2_ fixation and total chlorophyll (Chl) concentration in leaves of paddy rice (Ali et al., 2020). Biochar application to soil may have positive or no impact on different physiological indicators because of the difference of soil, biochar and different elements. For instance, biochar application to soil decreased leaf chlorophyll content in rice crop grown in upland nutrient poor soils (Wang et al., 2020), whereas Younis et al. (2015) reported an enhancement in transpiration rate, photosynthetic rate, and sub-stomatal CO_2_, as well as the concentration of chlorophylls. However, still there is lack of knowledge and need to weigh the photosynthetic traits such as photosynthetic rate (Pn), stomatal conductance (gs) and transpiration rate (Tr) for understating the effect of biochar on plant physiology in paddy soil.

Low N availability is a key limitation to crop production and can diminish yields by up to 50% (DeFries et al., 2012). Healthier growth and greater crop production require greater amounts of N fertilizer (Rennenberg & Dannenmann, 2015), and this requirement may increase up to threefold within a matter of days. However, plants may consume only half of the applied fertilizer (Schroeder et al., 2013), and the remainder leaches into groundwater or is emitted to the atmosphere, posing a significant threat to the environment and human health (Cameron et al., 2013). To enhance NUE and decrease the hazards associated with excess N use, sustainable agricultural techniques such as selecting crop-specific chemical fertilizers, adding organic amendments, and minimizing losses of nitrate (NO_3_^−^) are obligatory. To obtain a good yield, maximum leaf nitrogen concentration is necessary for proper utilization of resources. Hence, it is important to measure the amount of N taken up from contrasting N sources as a fraction of total leaf nitrogen at different growth stages provides information on the contribution of different fertilizers and their interactions with biochar. Thus, it was hypothesized that biochar addition will improve soil nutrient and water availability can alter plant morpho-physiological characteristics. Therefore, aim of the study was to determine the efficacy of traditional nitrogen fertilizers with or without biochar application on soil properties, root morphological traits, leaf physiological factors, and N concentration of paddy rice. Our findings will be useful for formulating new, sustainable management strategies to improve rice cultivation.

## 2 Materials and methods

### 2.1 Experimental site, soil, and temperature

Indoor pot experiments were conducted in the early (March–June) and late (August–November) seasons of 2019 at an experimental station of Guangxi University, Nanning, China, located at 22°49′20″N 108°17′04″E and an altitude of 75 m. This site encompasses warm, monsoon-influenced humid semitropical climate. The mean maximum and minimum temperatures of the greenhouse were 31.25 °C and 22.5 °C during the early season and 31.75 °C and 23 °C during the late season. The soil used in the experiment was collected from the top 20 cm horizon of the rice paddy at the research farm. It is classified as an ultisol (USDA taxonomy), and its initial physio-chemical properties were as follows: moisture 11.24%, bulk density 1.32 g cm^−3^, pH 5.92, soil organic carbon (SOC) 10.72 g kg^−1^, total nitrogen (Nt) 1.64 g kg^−1^, total phosphorus (P) 0.62 g kg^−1^, total potassium (K) 11.24 g kg^−1^, available N 132.31 mg kg^−1^, available P 23.15 mg kg^−1^, and available K 124.35 mg kg^−1^.

### 2.2 Crop management and experimental design

The rice cultivar Zhenguiai was grown in plastic trays, and seedlings of uniform size were transplanted to pots on 22 March and 15 August at a density of three hills per pot in the early and late season, respectively. The plastic PVC pots (50 cm height, 30 cm diameter, 706 cm^2^ surface area) contained air-dried and pulverized soil with or without biochar. The soil was adjusted to 50–60% of water holding capacity and was watered regularly before transplant to maintain the required moisture level. There were six treatments, each replicated nine times for a total of 54 pots arranged in a completely randomized design. To avoid experimental error, the pots were placed 20 cm apart. The treatment combinations were as follows: T1 (^15^N-urea), T2 (^15^N-urea + biochar), T3 (^15^N-ammonium nitrate), T4 (^15^N-ammonium nitrate + biochar), T5 (^15^N-ammonium sulfate), and T6 (^15^N-ammonium sulfate + biochar). The tested biochar used in the experiment was prepared from cassava straw as described in Ullah et al. (2020) and applied 10 days prior to transplant at the rate of 30 t ha^−1^ (211.8 g pot^−1^). Its basic chemical properties were pH 8.83, total carbon 674 g kg^−1^, sulfur 2.39 g kg^−1^, hydrogen 3.81 g kg^−1^, total N 5.43 g kg^−1^, total phosphorus 46.33 g kg^−1^, total potassium 48.33 g kg^−1^, and C:N ratio 112.24. Data were collected at three different growth stages: tillering, heading, and maturity. Therefore, three replicates were selected at each growth stage for destructive harvest (18 pots total).

All the ^15^N-labelled nitrogen fertilizers were applied at the rate of 315 kg ha^−1^ in three splits at the basal, tillering, and panicle initiation stages (5:3:2). The fertilizers included urea (U) at 4.83 g pot^−1^ with ^15^N enrichme nt of 10.08%, ammonium nitrate (AN) at 6.35 g pot^−1^ with 10.09% ^15^N enrichment, and am monium sulfate (AS) at 10.39 g pot^−1^ with 10.16% ^15^N enrichment. P was applied at the rate of 90 kg P ha^−1^ (3.17 g pot^−1^) as a basal dose to each pot. K was applied uniformly at a rate of 134 kg K ha^−1^ (1.57 g pot^−1^) in two splits (6:4) at the basal and tillering stages. The source of irrigation was tap water, and all pots were irrigated uniformly once in the morning and once in the evening to maintain the soil moisture level. Standard agronomic practices were performed similarly for all treatments throughout the growing seasons.

### 2.3 Sampling and measurement

Data were recorded at three growth stages in each season: tillering (23 April and 23 September), heading (13 May and 01 October) and maturity (12 June and 06 November). Leaf gas exchange parameters were measured at each growth stage before harvesting. For analysis of photosynthetic pigments and nitrogen metabolism enzymes, twelve fresh leaves were randomly selected from each treatment after harvesting, washed with distilled water, immediately placed in liquid nitrogen, and stored at − 80 °C. Root samples were collected and washed in tap water to remove clay. For measurement of total nitrogen and ^15^N, the remaining leaf samples were oven-dried at 70 °C for 48 hr to a constant weight. Soil samples were collected from the top 30 cm of soil at five randomly selected locations in each pot to measure soil chemical properties.

Soil pH was measured after shaking the soil with distilled water at a 1:2.5 (w/v) solid-to-water ratio for 1 hr. Values were recorded with a digital pH meter (Starter 2100 pH Bench, OHAUS). Soil organic carbon was measured using the oxidation method. Samples (0.5 g) were digested with 5 ml of 1 mol K_2_Cr_2_O_7_ and 5 ml of concentrated H_2_SO_4_, boiled at 175 ℃ for 5 min, and titrated with FeSO_4_ (Bao 2000). Total N was determined by weighing 1 g of plant tissue or 2 g of soil sample. The sample was placed in a digestion tube with 1 g of catalyst (potassium sulfate:copper sulfate:selenium powder 100:10:1), and concentrated H_2_SO_4_ was added (5 ml for the plant samples and 10 ml for the soil). The digestion tube was placed on a digester (X20A aluminum module automatic digester, Shanghai Shengsheng Automatic Analytical Instrument Co.) at 410 ℃ and digested until clear (2 hr for plant samples, 4 hr for soil samples). Sodium hydroxide (20–30 ml) was added, the distillate was absorbed with 2% boric acid solution in the digestion tube, and the indicator was methyl red bromocresol green. Samples were titrated with sulfuric acid and the volume recorded to determine N content using the following equation:

$$N\%=\frac{1.40\times N\times (V-V0)}{W}$$

where V_o_ (ml) is the blank titration volume, V (ml) is the sample titration volume, N is the standard acid equivalent concentration, and W (g) is the sample weight.

To measure ^15^N abundance, we added 1 ml (about 1 mg N ml^−1^) of the concentrated sample solution after Kjeldahl nitrogen determination to one side of a Y-shaped bottle and added 1 ml of lithium hypobromate solution to the other side. We then froze the solutions with liquid nitrogen; vacuumed to 5 × 10^−2^ Pa, defrosted, and mixed the reaction solution on both sides of the Y-shaped bottle to convert NH_4_^+^-N into N_2_. Under a high vacuum (1 × 10^−7^ m Bar), the generated N_2_ was ionized by an ion source, which transformed N_2_ into N_2_^+^, and the ion peak intensities of *m*/*z* 28 (^14^N^14^N) and 29 (^14^N^15^N) were recorded.

$$atom15N\%=\frac{1}{2\times R+1}\times 100$$

$$R=\frac{m}{z28}Ã\cdot \frac{m}{z29}$$

A mass spectrometer (Delta V Advantage IRMS, Thermo Fisher, USA) was used to measure ^15^N in leaf samples, and the measurement was performed as described in Huang et al. (2014). Available N in soil samples was measured using the alkaline potassium permanganate method (Sahrawat and Burford 1982). This procedure involves distilling the soil with an alkaline potassium permanganate solution and measuring the liberated ammonia by titration with sulfuric acid.

A number of root morphological traits were measured with an Epson Expression 10000XL scanner and root analysis software (WinRHIZO Pro v2009c, Regent Instruments, Quebec, Canada): total root length (TRL, m hill^−1^), total root surface area (TRSA, m^2^ hill^−1^), total average root diameter (TARD, mm hill^−1^), and total root volume (TRV, m^3^ hill^−^^1^). Leaf gas exchange parameters were measured at tillering, heading, and maturity in both seasons and included stomatal conductance (*gs*), transpiration rate (*E*), and net photosynthetic rate (*Pn*). Fully expanded leaves were randomly selected from each treatment and measured using a portable photosynthesis system (Li-6400, Li-COR Inc., Lincoln, NE, USA). The measurements were performed from 9:00 to 11:00 am when the plants were fully active.

Leaf Chl a and b were measured by the method of Porra et al. (1989). One gram of fresh leaf tissue was cut into small pieces, placed in a volumetric flask containing 10 ml of 80% acetone solution, and kept in the dark for 24 hr at 4 °C. The absorbance of the extracted solution was recorded at 663 and 645 nm using a UV spectrophotometer (Infinite M200, Tecan, Switzerland) to estimate Chl a and b concentrations by the standard method of Arnon (1949) expressed as mg g^−1^ fresh weight (FW).

C (Chl a) = 12.71 × A663 − 2.69 × A645

C (Chl b) = 22.88 × A645 − 4.67 × A663

The activity of nitrate reductase (NR) was measured by the method of Robin (1979) based on the total amount of nitrite formed. The absorbance of the reaction mixture was recorded at 540 nm, and the amount of NO_2_ formed was recorded using a standard calibration curve prepared from NaNO_2_ and expressed as μmol NO_2_ per gram FW per hour. The activity of GS was measured by the method of Lillo (1984). The absorbance of the reaction mixture was monitored at 540 nm, the enzyme activity was expressed as A540 nm g^−1^, and one unit of GS activity was defined as the amount of enzyme required to catalyze the formation of 1 mol glutamyl hydroxomate per hour at 37 °C. GOGAT activity was measured at 30 °C using the method of Singh and Srivastava (1986). The reduction in absorbance of the reaction mixture was recorded at 340 nm for 5 min. GOGAT activity was measured using a standard curve based on NADH, and the reaction mixture consisted of 10 mmol a-ketoglutarate, 1 mmol potassium chloride, 37.5 mmol Tris-HCl buffer (pH 7.6), 0.6 mmol NADH, 8 mmol L-glutamine, and 0.3 ml enzyme. One unit of GOGAT activity was defined as the amount required to reduce 1 μmol NADH per minute at 30 °C.

### 2.4 Statistical analysis

Data collected from all treatments were subjected to ANOVA. Mean separation was performed for significant ANOVA tests by the LSD method (*P* < 0.05) using Statistix 8.1. For correlation analysis, Correlations (Pearson) was used to evaluate the relationships between soil chemical properties and root morphological characteristics. Sigmaplot 12.0 and Microsoft Excel were used to create graphs and tables, respectively.

## 3 Results

### 3.1 Soil chemical properties

Regardless of N source, the addition of biochar significantly enhanced soil chemical properties (Table 1). In the early season, soil pH of the biochar treatments T2, T4 and T6 increased by 4.81%, 5.03% and 5.20% compared with their corresponding biochar-free treatments T1, T3 and T5. In the late season, soil pH increased by 5.94%, 6.56% and 6.21% in the biochar treatments. An average maximum pH of 6.23 was recorded in the biochar treatments, whereas the minimum pH was 5.90 in the biochar-free treatments in both seasons. Moreover, biochar addition caused a significant increase in SOC content in both seasons. SOC increased by 46.47%, 47.53% and 53.45% in the early season and by 52.45%, 51.57% and 55.81% in the late season in treatments T2, T4 and T6 relative to T1, T3 and T5. Additionally, the combination of biochar with synthetic N sources increased the soil N concentration by 4.26%, 4.57% and 4.95% in the early season and 6.61%, 5.28% and 4.43% in the late season relative to the corresponding N-only treatments. A similar trend was observed for available nitrogen in both seasons: the concentration was 30.40%, 28.72% and 24.36% higher in the early season in T2, T4 and T6, and 30.95%, 20.73% and 29.97% higher in the late season. Throughout the rice growth stages, there were significant differences in soil chemical properties among N sources applied with and without biochar (*P* < 0.05).

**Table 1:** Soil chemical properties under ^15^N-labelled fertilizers application with or without BC. **Note:** T1; ^15^N-U 315 kg N ha^−1^, T2; ^15^N-U 315 kg N ha^−1^ + BC 30 t ha^−1^, T3; ^15^N-AN 315 kg N ha^−1^, T4; ^15^N-AN 315 kg N ha^−1^ + BC 30 t ha^−1^, T5; ^15^N-AS 315 kg N ha^−1^, T6; ^15^N-AS 315 kg N ha^−1^ + BC 30 t ha^−1^.Values assigned with different letters within a column are significantly different at p < 0.05. (*n* = 3) n; number of samples.

Treatments	pH	SOC (g kg^−1^)	N*t* (g kg^−1^)	N*a* (mg kg^−1^)
Early season				
T1	5.89b	11.74b	1.57b	163.33b
T2	6.18 a	17.20 a	1.64 a	213.00 a
T3	5.89b	11.95b	1.57b	152.00b
T4	6.19 a	17.63 a	1.64 a	195.67 a
T5	5.89b	11.31b	1.57b	158.67b
T6	6.20 a	17.36 a	1.65 a	197.33 a
Late season				
T1	5.90b	11.21b	1.54c	149.67b
T2	6.25 a	17.22 a	1.65 a	196.00 a
T3	5.89b	11.36b	1.56 bc	154.33b
T4	6.28 a	17.22 a	1.65 a	186.33 a
T5	5.90b	11.35b	1.53c	145.67b
T6	6.27 a	17.69 a	1.60 ab	189.33 a

### 3.2 Root morphological traits

Root morphological characteristics are important physiological indicators, as the acquisition of water and nutrients by the root system drives plant growth. Biochar amendment with different nitrogen fertilizers significantly improved rice root morphological traits in both the early and late seasons (Table 2). At the tillering stage, biochar application (treatments T2, T4 and T6) increased TRL by an average of 6.19% and 4.97%, TRSA by 6.31% and 4.99%, TARD by 4.44% and 6.36%, and TRV by 6.20% and 5.05% compared with the biochar-free treatments (T1, T3 and T5) in the early and late seasons, respectively. Maximum root growth was observed at the heading stage across both seasons. At heading, biochar application increased TRL by an average of 10.45% and 11.10%, TRSA by 11.07% and 11.24%, TARD by 10.45% and 11.10%, and TRV by 10.44% and 11.09% in the early and late seasons. A reduction in root morphological traits was observed at the maturity stage relative to the heading stage. Nonetheless, the effect of biochar was similar to that observed at other growth stages. At maturity, TRL was increased by 4.67% and 2.51% in the biochar treatments, TRSA by 4.68% and 2.51%, TARD by 4.67% and 2.51%, and TRV by 4.71% and 2.51% in the early and late seasons. Root parameters in the biochar treatments differed significantly from those in the biochar-free treatments. The biochar treatments exhibited an average maximum root length of 147.96 and 155.90 m hill^−1^, TRSA of 31.26 and 33.72 m^2^ hill^−1^, TARD of 0.43 and 0.46 mm hill^−1^, and TRV of 39.60 and 42.56 cm^3^ hill^−1^ in the early and late seasons, respectively.

**Table 2:** Changes in root morphological features at the tillering, heading, and maturity stages as affected by ^15^N-labelled fertilizers with or without biochar.

Treatments	TRL	(m hill^−1^)	TRSA	(m^2^ hill^−1^)	TARD	(mm hill^−1^)	TRV	(cm^3^ hill^−1^)
Season	Early	Late	Early	Late	Early	Late	Early	Late
Tillering								
T1	82.76b	88.76 d	20.29b	22.10b	0.32c	0.34 ab	25.04b	26.45c
T2	91.10 a	96.19 a	22.36 a	23.95 a	0.34 ab	0.36 a	27.57 a	28.71 a
T3	86.93 ab	89.33 cd	21.33 ab	22.24b	0.32c	0.33b	26.30 ab	26.63c
T4	90.96 a	94.02 ab	22.34 a	23.41 ab	0.33 bc	0.36 a	27.52 a	28.05 ab
T5	88.15 a	90.71 cd	21.63 ab	22.58 ab	0.34 ab	0.35 ab	26.67 a	27.04 bc
T6	91.54 a	91.89 bc	22.48 a	22.88 ab	0.35 a	0.35 ab	27.70 a	27.40 bc
Heading								
T1	131.89b	137.87b	41.57c	42.55b	0.50b	0.53b	49.80b	51.88b
T2	144.83 a	154.24 a	45.91b	47.67 a	0.55 a	0.59 a	54.66 a	58.05 a
T3	135.21b	142.85b	42.07c	44.11b	0.52b	0.54b	51.02b	51.48b
T4	149.46 a	153.58 a	45.09b	47.46 a	0.57 a	0.59 a	56.40 a	57.82 a
T5	134.77b	140.33b	41.34c	43.32b	0.51b	0.53b	50.86b	52.84b
T6	149.61 a	159.90 a	47.81 a	49.44 a	0.57 a	0.61 a	56.48 a	60.20 a
Maturity								
T1	117.11 bc	124.65 ab	30.90b	33.85 a	0.45b	0.48 a	42.08 a	45.03 a
T2	123.61 a	127.25 ab	32.63 a	34.55 a	0.47 a	0.48 a	44.46 a	45.95 a
T3	119.33 abc	125.38 ab	31.49 ab	34.05 a	0.45 ab	0.47 a	42.86 a	45.30 a
T4	123.52 a	129.09 a	32.59 a	35.06 a	0.47 a	0.49 a	44.41 a	46.65 a
T5	116.40c	124.12 ab	30.71b	33.71 a	0.44b	0.47 a	41.80 a	44.87 a
T6	122.17 ab	127.25 ab	32.23 ab	34.56 a	0.46 ab	0.49 a	43.84 a	45.98 a

**Note:** TRL—total root length, TRSA—total root surface area, TARD—total average root diameter, TRV—total root volume. (*n* = 3) n: number of samples at each growth stage.

### 3.3 Leaf gas exchange parameters

#### 3.3.1 Stomatal conductance (gs)

The interaction between biochar and nitrogen fertilizers was associated with significant differences in *gs* at the heading stage in the early and late seasons and at maturity in the early season. There was no significant effect at the tillering stage in either season or at maturity in the late season (Table 3). Maximum net *gs* was observed in the biochar treatments throughout the growing seasons. At the tillering stage, *gs* was 8.33% higher in T4 than in T3 in the early season and 17.10% higher in the late season. Similarly, at heading and maturity, *gs* was 10.81% and 13.81% higher in T2 than in T1 in the early season and 13.83% and 6.91% higher in the late season. There were no significant differences among T2, T4 and T6 across the growth stages in either season.

**Table 3:** Changes in leaf gas exchange attributes at the tillering, heading, and maturity stages as affected by ^15^N-labelled fertilizers with or without biochar.

Treatments	gs (mol	H_2_O m^−2^ s^−1^)	*E* (mmol H_2_O	m^−2^ s^−1^)	*Pn* (µmol	CO_2_ m^−2^ s^−1^)
Season	Early	Late	Early	Late	Early	Late
Tillering						
T1	0.38 ab	0.40 ab	6.98 a	7.28c	23.42c	29.47c
T2	0.38 ab	0.44 ab	7.43 a	7.58b	25.02b	31.20 ab
T3	0.36b	0.38b	7.00 a	7.85 a	23.85c	29.60c
T4	0.39 a	0.45 a	7.33 a	7.57b	25.12 ab	32.57 a
T5	0.37 ab	0.42 ab	6.67 a	7.44 bc	23.82c	30.35 bc
T6	0.40 a	0.44 ab	7.38 a	7.63 ab	26.18 a	31.88 a
Heading						
T1	0.48c	0.48c	5.79c	6.82 ab	26.41 bc	27.88c
T2	0.54 a	0.52 a	6.72 a	7.08 a	27.64 ab	30.38 a
T3	0.48c	0.48c	5.77c	6.13b	26.71 abc	28.32c
T4	0.53 ab	0.53 a	6.49 ab	7.28 a	28.10 ab	30.17 ab
T5	0.49 bc	0.49 bc	5.92 bc	6.23b	25.28c	28.67 bc
T6	0.53 a	0.51 ab	6.86 a	7.06 a	28.90 a	30.05 ab
Maturity						
T1	0.24c	0.30 a	4.50c	4.66 ab	20.55 ab	19.49 a
T2	0.28 ab	0.32 a	5.22 ab	5.11 ab	22.02 a	21.59 a
T3	0.24c	0.29 a	4.43c	4.77 a	19.19 ab	20.71 a
T4	0.28 ab	0.32 a	4.95 bc	4.78 ab	21.89 a	22.18 a
T5	0.25 bc	0.28 a	4.46c	4.55b	18.10b	20.46 a
T6	0.28 a	0.31 a	5.86 a	4.96 ab	21.58 a	22.53 a

**Note:** gs; stomatal conductance, *E*; transpiration rate and *Pn;* photsynthetic activity. (*n* = 3) n; number of samples at each growth stage.

#### 3.3.2 Transpiration rate (E)

Transpiration rate (*E*) was significantly affected by the combined application of biochar and N fertilizer in both seasons. Overall, (*E*) was highest at tillering and decreased linearly with growth stage, reaching a minimum at maturity (Table 3). In the early season, there was no significant effect of treatment on *E* at tillering, whereas in the late season *E* was significantly higher by 3.69% in T3 than T4. Also, T3 did not differ statistically from T6. Across the seasons, *E* was increased by an average of 9.91%, 15.59% and 14.51% at heading and 12.75%, 6.04% and 20.09% at maturity in the biochar treatments T2, T4 and T6 relative to their corresponding treatments T1, T3 and T5. The rate of transpiration was higher in the late season than in the early season.

#### 3.3.3 Net photosynthesis (Pn)

Photosynthetic activity was significantly affected by the interaction of biochar and N sources at tillering and heading in both seasons, whereas there was no significant effect at maturity (Table 3). Treatments with added biochar showed high photosynthetic activity throughout the growing seasons. Across both seasons, *Pn* averaged 6.34%, 7.66% and 7.48% higher at tillering, 6.82%, 5.85% and 9.56% higher at heading, and 8.95%, 10.58% and 14.67% higher at maturity in the biochar treatments (T2, T4 and T6) than in their corresponding biochar-free treatments (T1, T3 and T5). *Pn* was higher at heading in the early season but higher at tillering in the late season. There were no significant differences (*P* < 0.05) among the biochar treatments (T2, T4 and T6) or the biochar-free treatments (T1, T3 and T5) at the heading and maturity stages.

### 3.4 Photosynthetic pigments

#### 3.4.1 Chlorophyll *A*

The effects of biochar amendment with nitrogen fertilizers on Chl a concentrations in rice leaves at different growth stages are shown in Table 4. The biochar treatments T2, T4 and T6 had higher Chl a concentrations (*P* < 0.05) in both seasons than the biochar-free treatments T1, T3 and T5. In the early season, the Chl a concentration was 15.90% higher at tillering, 29.76% higher at heading, and 2.28% higher at maturity in T2 than in T1. Likewise, in the late season, the Chl a concentration was 9.76% higher at tillering, 10.99% higher at heading, and 17.75% higher at maturity in T2 than in T1. There were no significant differences among treatments T2, T4 and T6 in either season. Biochar had a greater effect on Chl a concentration in the early season (average concentration 2.85 mg g^−1^) than in the late season (2.77 mg g^−1^). Notably, the combined biochar and fertilizer treatments produced significantly higher Chl a concentrations than their corresponding controls (*P* < 0.05).

**Table 4:** Leaf chlorophyll content at the tillering, heading, and maturity stages as affected by ^15^N-labelled fertilizers with or without biochar.

Treatments	Chl a (mg g^−1^)	Chl a (mg g^−1^)	Chl b (mg g^−1^)	Chl b (mg g^−1^)
Season	Early	Late	Early	Late
Tillering				
T1	3.12b	3.18b	3.14c	2.90c
T2	3.62 a	3.49 a	3.43 a	3.07b
T3	3.03b	3.18b	3.11c	2.91c
T4	3.62 a	3.48 a	3.50 a	3.20 a
T5	2.94b	3.17b	3.19 bc	3.04b
T6	3.54 a	3.51 a	3.40 ab	3.20 a
Heading				
T1	2.52c	2.73b	2.74b	2.54b
T2	3.27 a	3.03 a	3.05 a	2.82 a
T3	2.57 bc	2.77b	2.78b	2.55b
T4	3.00 a	2.97 a	3.00 a	2.79 a
T5	2.55c	2.78b	2.74b	2.57b
T6	2.95 ab	3.08 a	3.03 a	2.86 a
Maturity				
T1	1.90 ab	1.54b	1.92b	1.64b
T2	1.94 a	1.81 a	2.08 ab	1.80 a
T3	1.78b	1.54b	1.87b	1.74 ab
T4	1.88 ab	1.76 a	2.11 ab	1.85 a
T5	1.83 ab	1.59b	1.90b	1.67b
T6	1.87 ab	1.82 a	2.38 a	1.81 a

**Note:** Chl-a, chlorophyll-a; Chl-b, chlorophyll-b. (*n* = 3) n: number of samples at each growth stage.

#### 3.4.2 Chlorophyll *B*

The effects of biochar and nitrogen fertilizers on Chl b concentrations in leaves are presented in Table 4. The concentration of Chl b was significantly higher in biochar treatments in both seasons (*P* < 0.05). In both seasons, the Chl b concentration was 9.41% higher in T4 than in T3 at tillering. The maximum Chl b concentration at heading was observed in T2 (3.05 mg g^−1^) in the early season and in T6 (2.86 mg g^−1^) in the late season. The minimum concentration was observed in T1, followed by T3 and T6. At maturity, the Chl b concentration averaged 7.74% higher in T2, T4 and T6 than in T1, T3 and T5. Moreover, the Chl b concentration was significantly higher (*P* < 0.05) in the biochar treatments than in the biochar-free treatments, although there were no significant differences among the biochar treatments (T2,T4 and T6) or the biochar-free treatments (T1,T3 and T5) across the seasons.

### 3.5 Activities of N metabolism enzymes

#### 3.5.1 Nitrate reductase (NR)

**Fig. 1:**
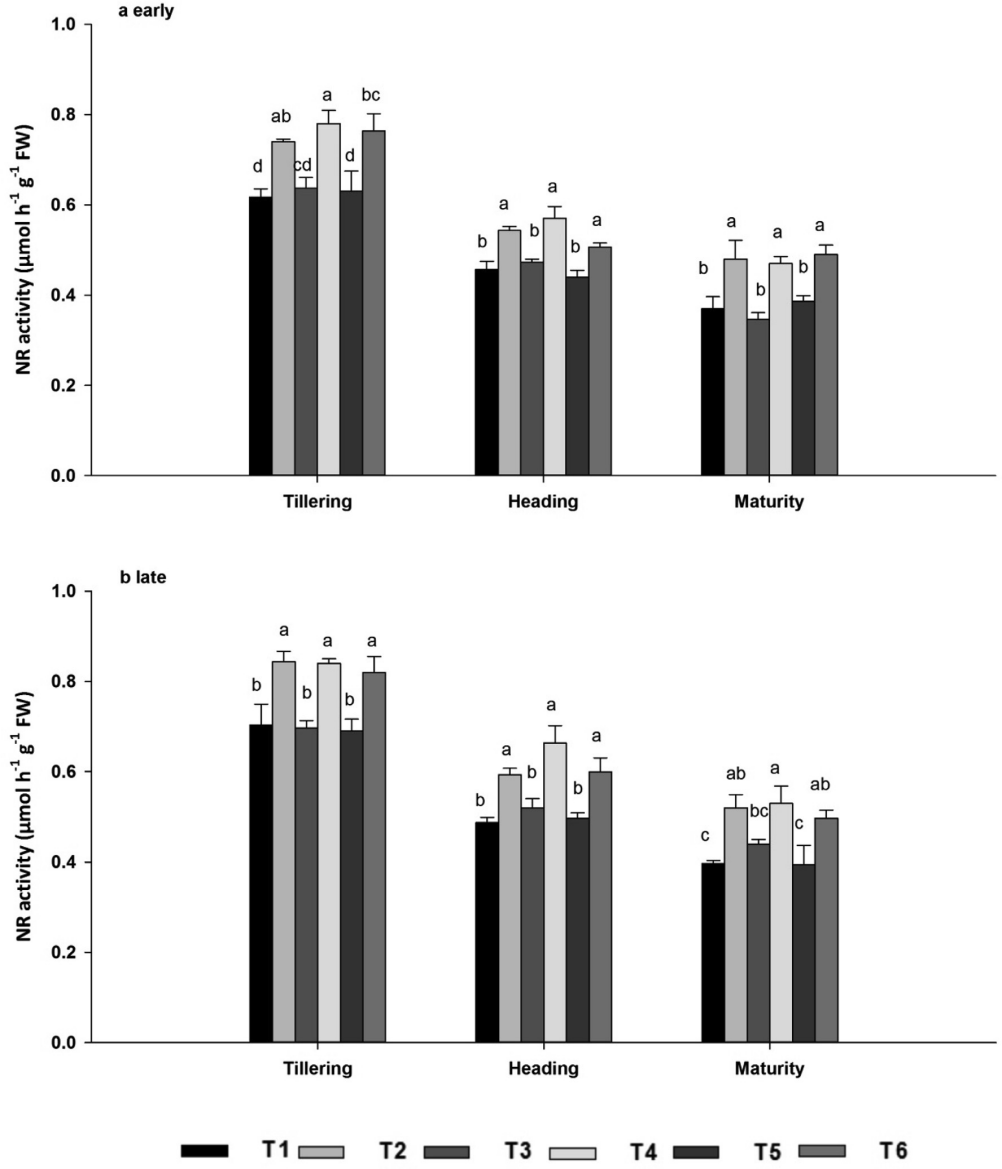
NR enzyme activities during the early (a) and late (b) seasons, at the tillering, heading, and maturity stages as affected by ^15^N-labelled fertilizers with or without biochar. Note: T1; ^15^N-U 315 kg N ha^−1^, T2; ^15^N-U 315 kg N ha^−1^ + BC 30 t ha^−1^, T3; ^15^N-AN 315 kg N ha^−1^, T4; ^15^N-AN 315 kg N ha^−1^ + BC 30 t ha^−1^, T5; ^15^N-AS 315 kg N ha^−1^, T6; ^15^N-AS 315 kg N ha^−1^ + BC 30 t ha^−1^. NR; Nitrate reductase. Vertical bars represent standard error, SE of the mean (*n* = 3), n; number of samples. Bars having different letters are significantly different from each other at P < 0.05.

NR, a molybdoenzyme, reduces nitrate to nitrite and facilitates protein synthesis in plants. The NR activity at tillering, heading and maturity was significantly affected (*P* < 0.05) by the combined application of nitrogen fertilizers and biochar (Fig. 1A–B). The activity of NR decreased markedly with leaf senescence: in both seasons, it was highest at tillering, declined at heading, and reached a minimum at maturity. In both seasons, NR activity was 19.5%, 21.53% and 20% higher at tillering, 20.53%, 23.99% and 20.45% higher at heading, and 30.41%, 28.05% and 26.53% higher at maturity in T2, T4 and T6 compared with T1, T3 and T5, respectively.

#### 3.5.2 Glutamine synthetase (GS)

**Fig. 2:**
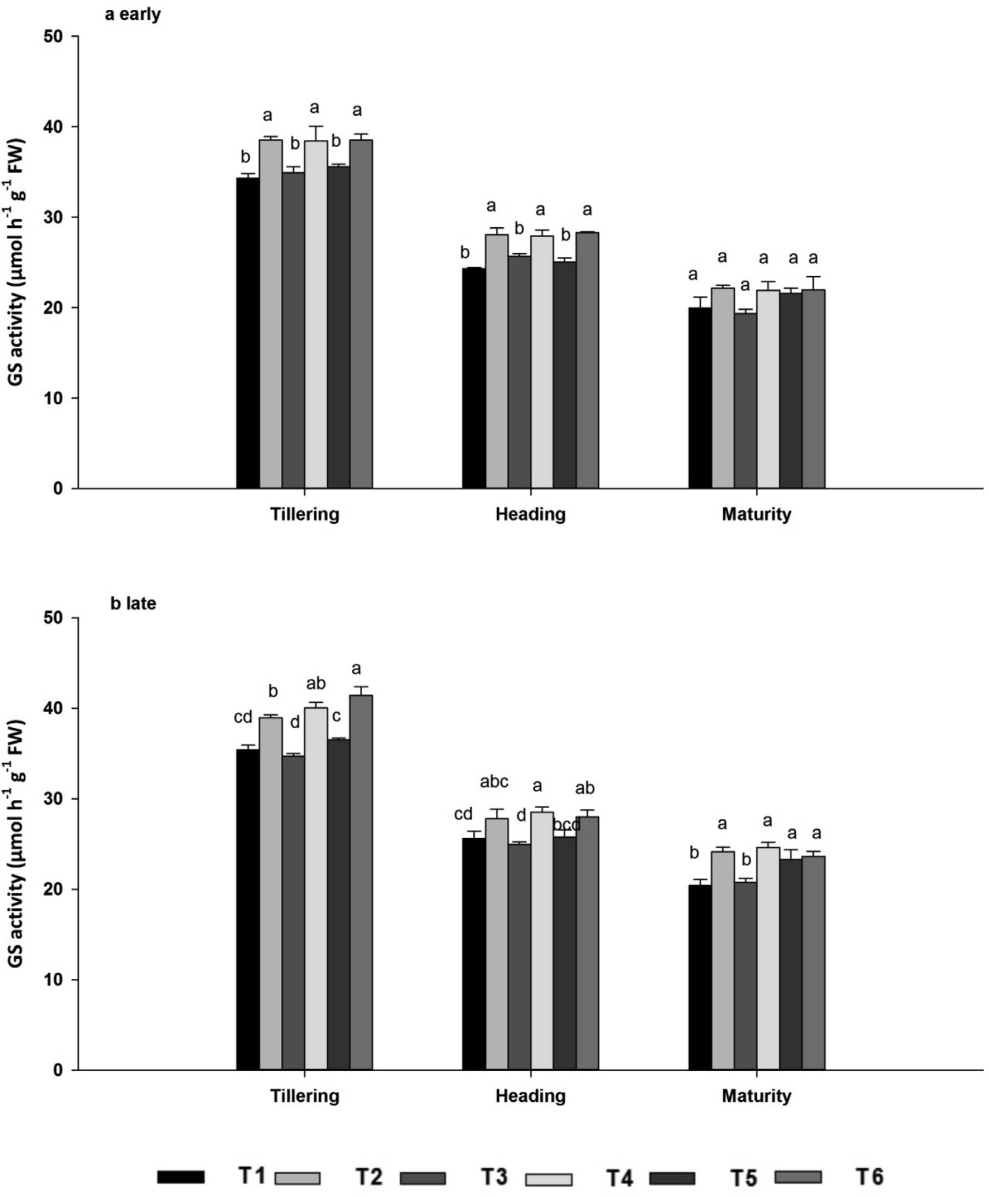
GS enzyme activities during the early (a) and late (b) seasons, at the tillering, heading, and maturity stages as affected by ^15^N-labelled fertilizers with or without biochar. Note: GS; Glutamine synthetase. Vertical bars represent standard error, SE of the mean (*n* = 3), n; number of samples. Bars having different letters are significantly different from each other at P < 0.05.

Glutamine synthetase plays a crucial role in the metabolism of nitrogen by catalyzing the condensation of glutamate and ammonia to form glutamine. Similar to NR activity, GS activity was highest at tillering, declined at heading, and reached a minimum at maturity. GS activity was significantly affected by the combined application of N fertilizers and biochar, regardless of N source (Fig. 2A–B). Across the seasons, GS activity was 11.17%, 12.82% and 10.86% higher at tillering, 11.87%, 11.44% and 10.87% higher at heading, and 14.66%, 16.03% and 1.68% higher at maturity in the biochar treatments T2, T4 and T6 than in the biochar-free treatmentsT1, T3 and T5, respectively. There were no significant differences among T2, T4 and T6 (*P* < 0.05) or among T1, T3 and T5 in either season.

#### 3.5.3 Glutamine oxoglutarate aminotransferase (GOGAT)

**Fig. 3:**
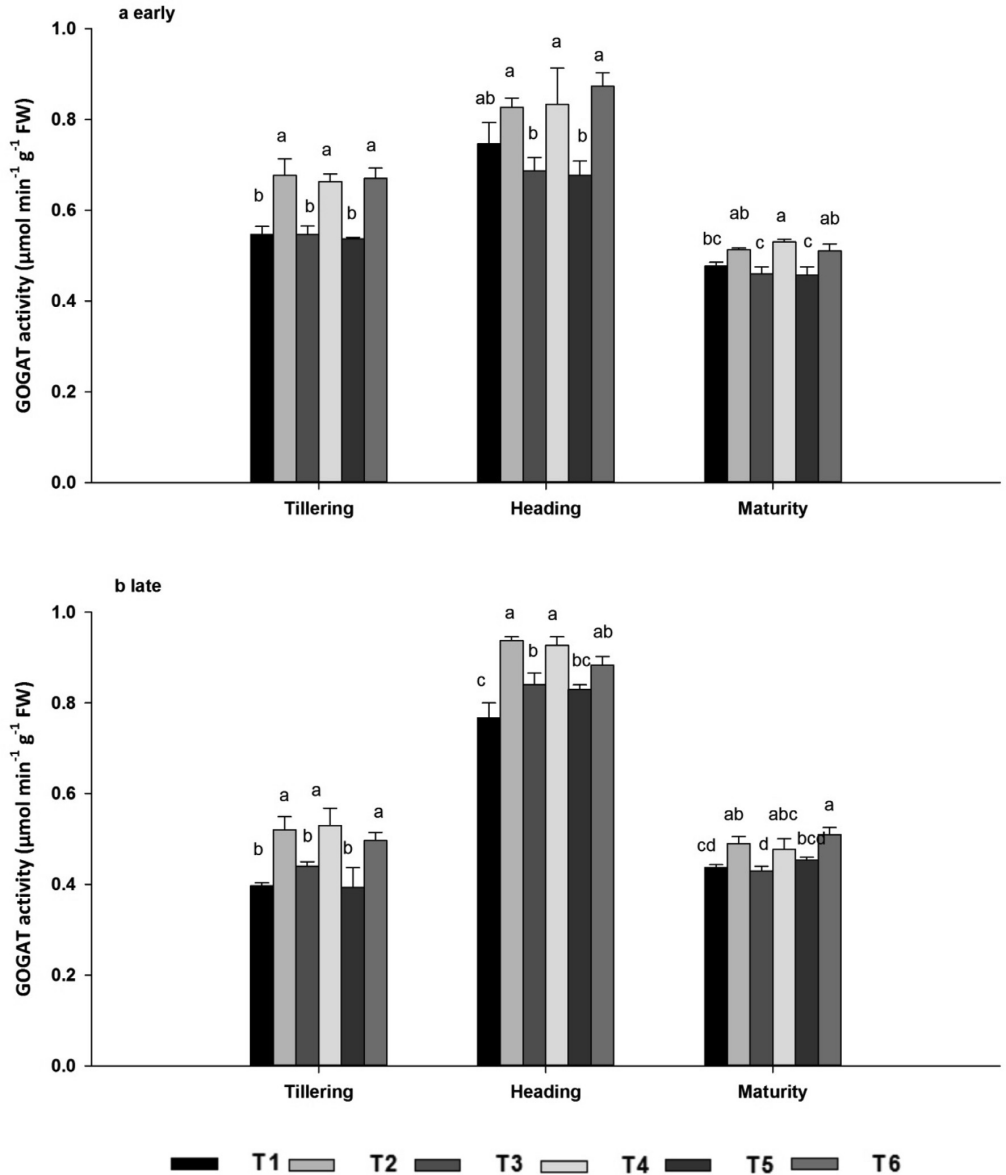
GOGAT enzyme activities during the early (a) and late (b) seasons, at the tillering, heading, and maturity stages as affected by ^15^N-labelled fertilizers with or without biochar. Note: GOGAT; Glutamine oxoglutarate aminotransferase. Vertical bars represent standard error, SE of the mean (*n* = 3), n; number of samples. Bars having different letters are significantly different from each other at P < 0.05.

GOGAT synthesizes glutamate from glutamine and α-ketoglutarate; together with glutamine synthetase, it plays a vital role in nitrogen assimilation. A quadratic trend was observed in GOGAT activity: it reached a maximum at heading, was intermediate at tillering, and declined to a minimum at maturity in both seasons (Fig. 3A–B). Biochar application with N fertilizers produced maximum rates of GOGAT enzyme activity (0.87 µmol min^−1^) at the heading stage in both seasons. Moreover, across both seasons, GOGAT activity was 27.43%, 20.89% and 25.78% higher at tillering, 22.97%, 15.82% and 15.63% higher at heading, and 9.95%, 12.89% and 12.08% higher at maturity in T2, T4 and T6 compared with T1, T3 and T5, respectively. There were no significant differences (*P* < 0.05) among T2, T4 and T6.

### 3.6 Leaf nitrogen concentrations

#### 3.6.1 Total N concentrations in leaves

**Fig. 4:**
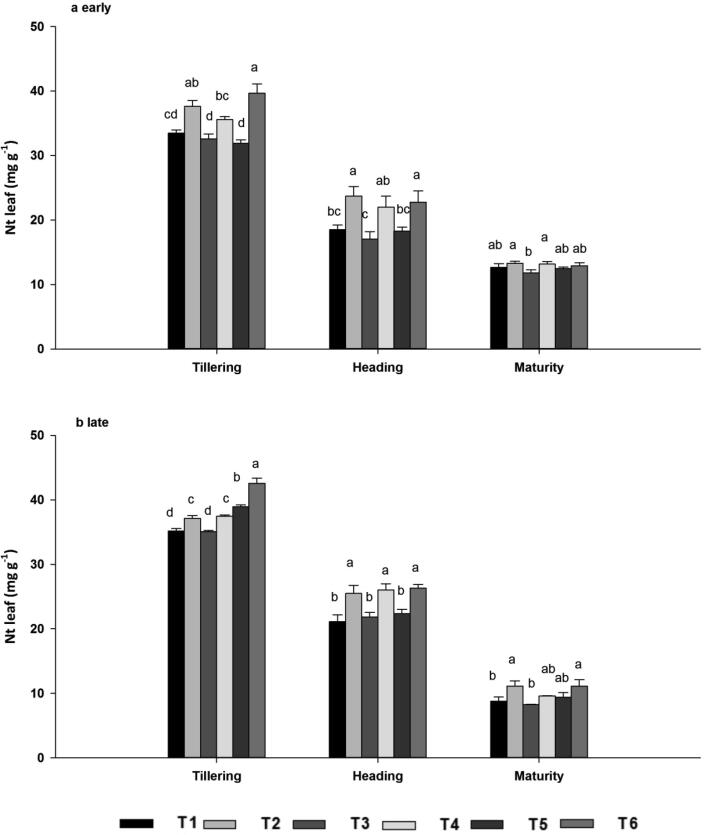
Leaf total nitrogen concentrations (N*t*) during the early (a) and late (b) seasons, at the tillering, heading, and maturity stages as affected by ^15^N-labelled fertilizers with or without biochar. Note: Vertical bars represent standard error, SE of the mean (*n* = 3), n; number of samples. Bars having different letters are significantly different from each other at P < 0.05.

Biochar addition with nitrogen fertilizers significantly enhanced leaf N concentration in both early and late rice. However, N concentration decreased with leaf senescence because of translocation from the leaves to the panicles. Therefore, the effect of biochar on leaf N was not significant at maturity in early rice, although it was significant in late rice (Fig. 4A–B). Higher leaf N concentration in response to biochar addition was observed at tillering in T6 (39.64 mg g^−1^) in the early season and at heading in T6 (42.56 mg g^−1^) in the late season, and these values did not differ significantly from those of T2 and T4. Moreover, there were no significant differences in leaf N among the biochar-free treatments T1, T3 and T5. Across the seasons, total N content was 8.96%, 7.97% and 16.77% higher at tillering, 24.37%, 23.92% and 21.06% higher at heading, and 15.77%, 13.81% and 10.78% higher at maturity in T2, T4 and T6 compared with T1, T3 and T5, respectively. Leaf N concentration was higher in the late season than in the early season.

#### 3.6.2 ^15^N concentrations in leaves

**Fig. 5:**
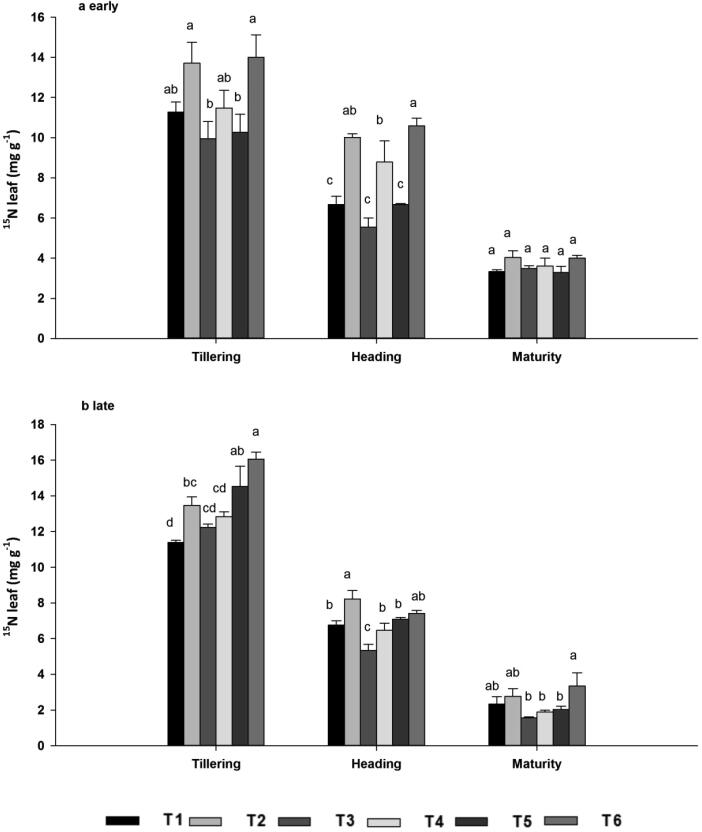
Leaf ^15^N concentrations during the early (a) and late (b) seasons, at the tillering, heading, and maturity stages as affected by ^15^N-labelled fertilizers with or without biochar. Note: Vertical bars represent standard error, SE of the mean (*n* = 3), n; number of samples. Bars having different letters are significantly different from each other at P < 0.05.

Biochar application significantly (*P* < 0.05) increased leaf ^15^N concentration regardless of N source at the tillering and heading stages, whereas it had no significant effect at maturity in either season (Fig. 5A–B). Compared with the unamended treatments T1, T3 and T5, leaf ^15^N concentration in the amended treatments T2, T4 and T6 increased by 19.95%, 10.06% and 23.41% at tillering, 35.82%, 39.74% and 31.66% at heading, and 20.09%, 11.89% and 43.26% at maturity across both seasons. Leaf ^15^N concentration decreased linearly as a result of N translocation from leaves to panicles. Across the seasons, the average ^15^N concentration in the biochar treatments was 13.59 mg g^−1^ at the tillering stage, 8.58 mg g^−1^ at the heading stage and 3.27 mg g^−1^ at maturity, whereas that of the biochar-free treatments was 11.60 mg g^−1^ at tillering, 6.34 mg g^−1^ at heading, and 2.67 mg g^−1^ at maturity. However, there were significant differences among the N sources with and without biochar.

### 3.7 Correlation of root morphological traits with soil chemical properties

The relationships between rice root morphological attributes and soil chemical properties across the seasons are presented in Table 5. Root morphological features and TRL, TRSA, TARD, and TRV were significantly positively correlated with SOC, N*t* and N*a* across the seasons (*P* < 0.001). These findings indicate that increases in paddy soil carbon and nitrogen stocks directly influence root morphological properties.

**Table 5:** Correlation coefficient of soil chemical properties with root morphological features across the seasons.

	pH	SOC	TN	AN	TRL	TRSA	TARD
SOC	0.9846^***^						
TN	0.9433^***^	0.9386^***^					
AN	0.9360^***^	0.9362^***^	0.9290^***^				
TRL	0.9339^***^	0.9388^***^	0.8895^***^	0.8530^***^			
TRSA	0.9423^***^	0.9455^***^	0.8997^***^	0.8859^***^	0.9478^***^		
TARD	0.9589^***^	0.9615^***^	0.9048^***^	0.9001^***^	0.9730^***^	0.9607^***^	
TRV	0.9517^***^	0.9498^***^	0.9159^***^	0.8926^***^	0.9487^***^	0.9542^***^	0.9825^***^

**Note:** For correlation analysis, Correlations (Pearson) was used to evaluate the relationships between soil chemical properties and root morphological characteristics.*** represents statistical significance at p < 0.001 and data were averaged of both seasons, as shown same behavior across the seasons. (*n* = 9).

## 4 Discussion

### 4.1 Impact of biochar and nitrogen fertilizers on soil chemical properties

In the current study, combined application of biochar and N fertilizer significantly improved soil chemical properties (Table 1). Soil pH is a crucial indicator of the biochar effect on soil chemical properties, and the increase in pH observed in our findings may have been due to the alkaline nature of the cassava straw biochar. As reported in literature (Mukherjee et al., 2014), that addition of oak wood biochar (with an initial pH of 9.4) significantly increased pH by 0.4 units. Several studies have reported that soil pH increases due to biochar incorporation, particularly on acidic soils, and that biochar application ameliorates the supply of nutrients to plants and improves carbon sequestration (Atkinson et al., 2010, Vassilev et al., 2013). This directed that the additions of biochar with inorganic N fertilizer are effective measures to improve the pH of paddy soil. Biochar plays an essential role in carbon sequestration, which is facilitated by the adsorption capacity of organic molecules (McBratney et al., 2014). In the present study, the addition of biochar to paddy soils augmented SOC compared with non-amended treatments. The possible reasons might be an enhancement in oxidisable organic carbon (OC) concentrations, probably due to augmented plant-derived organic matter (OM) application with N sources, as well as the release of relatively simpler forms of OC in biochar during its degradation in the soils. Consistent with our results, Huang et al., 2017a, Huang et al., 2017b, Xi et al., 2015 reported that straw return increased the fraction of labile organic matter and that SOC increased significantly after straw return. Li et al. (2018) reported that enrichment in SOC after biochar addition is due to the stable structure inside the biochar, which suppresses the surface oxidation of organic C, improves the stability of SOC against microbial decomposition, and thereby improves soil carbon content. Similarly, the N content of biochar-amended soil was significantly higher than that of un-amended soil in both early and late seasons. Many studies have shown that biochar addition increases soil nutrient content (Ali et al., xxxx, Liang et al., 2014). This is due in part to the direct incorporation of nutrients from the biochar such as N, P and K (Enders et al., 2012) and in part to reductions in runoff and leaching (Laird et al., 2010). In this investigation, higher N stock was observed in biochar treatments, regardless of N source. Moreover biochar applied treatments performed better than only N fertilizer treatments, in term of soil available nitrogen. This may be because biochar has much porous structure and higher adsorption capacity for inorganic nutrients than untreated soil, which offers a suitable habitat for microbial community (Allison et al., 2014, Zavalloni et al., 2011). Biochar can improve the absorption and retention of N, decrease N runoff, and enrich N transformation, resulting in higher N availability (Yu et al., 2014).

### 4.2 Impact of biochar and nitrogen fertilizers on root growth

Roots are the primary source of water and nutrient acquisition in plants, producing organic acids, amino acids and hormones (Xiang et al., 2017). Furthermore, root morphological traits are closely linked to the growth and development of the plant (Yu et al., 2019). Cultivation techniques, soil physicochemical properties, and fertilizer amendment can easily affect root morphology (Jha et al., 2017). In the present study, biochar altered root growth environment and improved root length, surface area, diameter and volume, regardless of N source in both seasons and at all growth stages (Table 2). Our results are consistent with those of Changxun et al. (2016), which showed that biochar increased root volume, length, and surface area of *Poncirus trifoliata* (L.) Raf. seedlings growing in Gannan acidic red soil. Our results also revealed that biochar application enhanced root growth in a linear manner from tillering to heading, whereas at maturity root growth was somewhat reduced, which may be ascribed to that the biochar degradation may have been delayed in later growth stages, and the growth of soil microorganisms was reduced. However, overall root performance was better with than without biochar (Table 2). This is consistent with the previous investigation of Yang et al. (2015), who reported that applying biochar enhanced root morphology during the seedling stage of a sugarcane cultivar. Changes in root morphological traits between biochar applied and non-applied treatments could be explained by the fact that fate of root are associated with fluctuations in soil physicochemical properties, and enhanced soil properties have a profound influence on root growth (Ezawa et al., 2002). In finding of Elad et al. (2010), soil applied with biochar produced organo-mineral complexes that improved nutrient availability and presence in the soil. Biochar amendment also enriched microbial community complexes and strengthened systemic plant defenses against soil pathogens, which consequently increased root biomass and plant health with little cost to the environment (Elad et al., 2010). Overall, these results showed that the joint application of biochar and N has the potential to improve plant growth efficiency. Razaq et al. (2017) reported that plant nitrogen requirement is lower in the seedling period but increases markedly during subsequent growth stages. The results indicated that quantity of N in the soil regulates the plant’s growth, and more fertilizer is required to maintain maximum growth at later growth stages. In the present study, possible increased in root growth may have also contributed to higher nitrogen availability in treatments where biochar was applied in combination with N fertilizer, and, consistently, a positive correlation was noted between root traits and soil chemical properties (Table 5). Similarly, Xiang et al. (2017) reported that biochar fertilization improved root morphological attributes: root biomass (32%), volume (29%), surface area (39%), length (52%) and diameter (10%).

### 4.3 Impact of biochar and nitrogen fertilizers on leaf gas exchange attributes and chlorophyll content

A significant difference was recorded in leaf gas exchange attributes (stomatal conductance, transpiration rate and photosynthesis rate) as affected by fertilizer sources applied with and without biochar in both early and late seasons. Generally, biochar treatments were associated with higher photosynthesis, stomatal conductance and transpiration rate than biochar-free treatments. This was probably due to greater nutrient availability in the amended soil throughout the growing seasons, different mineralization process occurred under environmental conditions and healthier root system (Table 1, Table 2). In agreement with our findings Slavich et al., 2013, Wang et al., 2012 reported higher stomatol conductance reflect the presence of potassium and phosphorus in the biochar, which may be released at high levels into the soil through mineralization. Changes in soil water holding capacity (WHC), enrichment of the microbial community, and reduction in nutrient leaching may also be indirect effects of biochar amendment (Tian et al., 2018). Leaf photosynthetic capacity plays a significant role in the regulation of crop yield (Xu & Shen, 2002). In the current study, photosynthetic rates were higher in biochar-amended treatments than in the corresponding N-only treatments in both seasons, particularly at the heading stage. The probable reason might be higher availability of nitrogen contents can increases cell wall rigidity and photosynthetic capacity in plants (Chen et al., 2016). Moreover, the stomatal conductivity of water was associated with transpiration rate, results water in leaf to evaporate. Mechanism of stomatal conductivity played an efficient role in carbon assimilation for the photosynthesis process and water elimination of transpiration rate (Acatrinei, 2010). Hence, improvement in stomata conductance is known to enhance intercellular CO_2_ concentration followed to increase photosynthesis rate. These results suggest nitrogen supply improved leaf gas exchange attribute in biochar applied soil as compared to only fertilizers application. Similar results have been reported by other researchers (Abel et al., 2013, Agegnehu et al., 2017): biochar amendment enhances water holding ability and BD, resulting in greater stomatal conductance, more CO_2_ diffusion, improved net photosynthesis, and greater crop production. The most likely reasons for high photosynthetic activity in biochar treatments are improved transpiration and higher nitrogen content (Table 1, Table 2, Table 3). Photosynthesis is profoundly influenced by nitrogen availability (Xu et al., 2015) because 57% of leaf nitrogen is contained in the chloroplasts and used to synthesize photosynthetic components and allied enzymes (Xu et al., 2012).

Moreover, the increase in *Pn* may be explained by higher leaf *g*s and *E* following biochar amendment (Table 3). Greater *g*s and *E* may be linked to improved soil water holding capacity associated with the porous physical structure of the biochar (Laghari et al. 2015). In line with the results of Xu et al. (2012), low nitrogen may cause decreased photosynthetic activity due to poor carboxylation, consistent with elevated intercellular CO_2_ concentrations. In this study all the fertilizer rates were similar, but the contrasting N sources with and without biochar may influence the N availability to the plant, leads to variation in leaf gas exchange attributes. Thus, results established that higher photosynthetic activity was generated by organic -mineral composites, which was higher than those produced by synthetic fertilizers were applied at the same rate. Total Chl consists mainly of Chl a and Chl b: Chl a is concentrated in the photosystems, whereas Chl b is most abundant in the light harvesting complexes (Venema et al., 2000). In the current study, higher leaf Chl a and b concentrations were observed in the biochar treatments in both seasons (Table 4), probably reflecting the higher N availability and leaf enzymatic activities in the biochar treatments (Fig. 1, Fig. 2, Fig. 3, Fig. 4, Fig. 5). Biochar is known to improve water and nutrient availability to roots, substantially increasing pigment synthesis and N absorption in plant leaves (Speratti et al., 2018). Plants release ethylene under nutritional stress, and if this ethylene reaches the chloroplast, membrane lipids are reduced and expression of the chlorophyllase gene is upregulated (Michaud & Jouhet, 2019). Chlorophyllase then degrades Chl, ultimately resulting in chlorosis (Shu et al., 2016). In the present work, Chl levels were lower in treatments without biochar, perhaps reflecting the synthesis of ethylene. Improvements in Chl concentration due to biochar application provided additional evidence that biochar may be useful for increasing photosynthesis and plant biomass.

### 4.4 Impact of biochar and nitrogen fertilizers on nitrogen metabolism enzymes

Nitrogen metabolism enzymes such as NR, GS, and GOGAT play a key role in plant N assimilation (Forde & Lea, 2007). During N assimilation, NR catalyzes the reduction of nitrate to nitrite with pyridine nucleotide and GS-GOGAT cycle is the key pathway of ammonia assimilation in plants, nearly 90 to 95% of NH_4_^+^ incorporation occurs through this cycle (Masclaux-Daubresse et al., 2006). In addition, N metabolism enzymes were measured to evaluate the differences for treatments and the maximum enzymes activity were recorded when biochar was combined with N fertilizer across all growth stages and seasons. The variation in enzymes activity between these treatments may be linked with dissimilarity in regulation of N transporter genes or N fluxes in roots (Glass, 1995). As reported by Farhang i-Abriz & Torabian, (2018) that biochar could improve soil N cycling by increasing nitrification, reducing NH_3_ volatilization, and promoting NH_4_^+^ accumulation by altering soil cation exchange capacity. In agreement with our findings Thomas & Gale, (2015) reported that biochar combined with N application promoted greater leaf N assimilation than N alone and was essential for delivering adequate substrates during grain filling and improving rice grain yield. Compared with tillering and heading growth stages, enzymatic activity decreased in the maturity stage. At maturity nitrogen accumulated in leaf were translocated to the sink (panicles) for grain formation (Xu et al., 2012). The minimum activity at maturity stage might be due to the photosynthesis virtually declines because of the hydrolysis of flag leaf cellular components into transport compounds with low C/N ratio to develop seed for their accumulation (Kichey et al., 2005). Consistent with our results, Farhangi-Abriz & Torabian (2018) stated that biochar improved soybean nodulation, N content, and N metabolism by enhancing N fixation and the activities of NR, GS, and GOGAT*.* N uptake was associated with enhanced activities of N metabolism enzymes. N concentrations were highest following joint application of N fertilizer and biochar across the seasons, both in terms of total nitrogen and ^15^N from labelled fertilizer (Fig. 4, Fig. 5). N taken up by roots is reduced and transferred to the shoots for assimilation (Xu,et al., 2012). During initial assimilation, N is converted into glutamine and glutamate, followed by the formation of amino acids, proteins, and other nitrogenous compounds (Pratelli & Pilot, 2014). The earliest stages of N assimilation are catalyzed by enzymes such as NR, GS, GOGAT, and GDH (Funayama et al., 2013). Thus, a well-synchronized system of N uptake and assimilation may be the base of the high N concentration in leaf in biochar applied treatments, which was elucidated by their maximum enzymatic activities (Fig. 1, Fig. 2, Fig. 3).

### 4.5 Impact of biochar and nitrogen fertilizers on leaf nitrogen concentration

Nutrient uptake efficiency reflects both adequate soil nutrient supply and the ability of plants to utilize the nutrients (Yang & Zhang, 2010). Biochar amendment had a significant effect on total nitrogen uptake from soil as well as N uptake from the labelled fertilizer. A synergistic effect of biochar and N fertilizer promoted N uptake, and total N was 31.8% and 14.54% higher in biochar vs. biochar-free treatments across all growth stages. Moreover, there were no significant differences among the biochar treatments or the biochar-free treatments. The lower leaf N concentration of non-applied treatments might be due to fragile root growth, low photosynthetic efficiency, poor N uptake and metabolism as showed in the results section, and these results are in line with the previous studies (Mu et al., 2016, Uribelarrea et al., 2009). Fertilizer N applied to rice paddies can be lost through denitrification, ammonia (NH_3_) volatilization, and leaching (Peng et al., 2006, Ullah et al., 2021). Biochar is used as a mitigation tool to reduce nitrogen losses, ultimately enhancing nitrogen availability to plants. The amount of N loss from cultivated soils due to denitrification differs enormously with crop management practices, soil physio-chemical properties, and environmental conditions, ranging from 0 to 70% of the fertilizer applied (Wu et al., 2013). It has been reported that the combination of biochar with synthetic fertilizers can reduce the activity of denitrifying bacteria, the activity of reductases that convert nitrite and nitrate to N_2_O (Yanai et al., 2007), and the activity of urease that is associated with N_2_O emissions (Wu et al., 2013) in agricultural soils. These effects may also be partly responsible for reduced N losses in biochar treatments in the present study. Biochar application to soils may significantly affect root morphology, thereby enhancing root uptake of nutrients (Prendergast-Miller et al., 2014). Similarly, Liu et al. (2013) reported that soil biochar amendment increased crop production by about 10%, resulting in higher N use efficiency. Our results showed that biochar application improved root morphological characteristics that may enhance N uptake by plants (Table 2). Iqbal et al. (2019) also reported strong relationships among dry matter accumulation, N uptake and root growth of rice in a pot experiment. N uptake from N fertilizer was improved because the application of biochar to the soil reduced N_2_O emissions and in turn increased N availability to plants (Case et al., 2015).

## 5 Conclusion

Here, we have established that biochar acts as a soil conditioner, enriching soil quality, improving root growth and development, promoting leaf gas exchange, increasing levels of photosynthetic pigments, and enhancing nitrogen concentration. Interestingly, the activities of N metabolism enzymes were positively correlated with N uptake, and their activity increased with higher N levels. Biochar also enhanced carbon sequestration and fertilizer efficiency. This study enabled us to investigate the response of different morpho-physiological parameters to contrasting nitrogen sources, and we found significant differences among the treatments. In term of leaf N concentration the fertilizer was ranked in order ammonium sulfate > urea > ammonium nitrate. The overall performance of N fertilizers applied with biochar was better than that of the fertilizers alone. Therefore, a combined application of N and biochar can be recommended as a sustainable strategy for increasing nitrogen uptake and soil fertility under controlled conditions. However, an additional large-scale approach will be necessary to obtain further information and serve as the basis for long-term, extensive rice cultivation.

**Funding:** This research was financially supported by the National Key Research and Development Project of China (2018YFD20030503).

## Declaration of Competing Interest

The authors declared that there is no conflict of interest.

## References

[b0005] Abel S., Peters A., Trinks S., Schonsky H., Facklam M., Wessolek G. (2013). Impact of biochar and hydrochar addition on water retention and water repellency of sandy soil. Geoderma.

[b0010] Acatrinei L. (2010). Photosynthesis rate, transpiration and stomatal conductance of vegetable species in protected organic crops. Lucrări Ştiinţifice.

[b0015] Agegnehu G., Srivastava A., Bird M.I. (2017). The role of biochar and biochar-compost in improving soil quality and crop performance: A review. Appl. Soil Ecol..

[b0020] Ali I, He L, Ullah S, Quan Z, Wei S, Iqbal A, Munsif F, Shah T, Xuan Y, Luo Y. Biochar addition coupled with nitrogen fertilization impacts on soil quality, crop productivity, and nitrogen uptake under double‐cropping system. Food and Energy Security.e208.

[b0025] Allison S.D., Chacon S.S., German D.P. (2014). Substrate concentration constraints on microbial decomposition. Soil Biol. Biochem..

[b0030] Arnon D.I. (1949). Copper enzymes in isolated chloroplasts. Polyphenoloxidase in Beta vulgaris. Plant physiology..

[b0035] Atkinson C.J., Fitzgerald J.D., Hipps N.A. (2010). Potential mechanisms for achieving agricultural benefits from biochar application to temperate soils: a review. Plant and.

[b0040] Bao S. (2000). Agro-chemical analysis of soil.

[b0045] Bruun E.W., Petersen C.T., Hansen E., Holm J.K., Hauggaard-Nielsen H. (2014). Biochar amendment to coarse sandy subsoil improves root growth and increases water retention. Soil use and management.

[b0050] Cameron K., Di H.J., Moir J. (2013). Nitrogen losses from the soil/plant system: a review. Annals of applied biology.

[b0055] Carrijo, D.R.; Lundy, M.E.; Linquist, B.A. Rice yields and water use under alternate wetting and dryingirrigation: A meta-analysis. Field Crops Res. 2017, 203, 173–180.

[b0060] Case S.D., McNamara N.P., Reay D.S., Stott A.W., Grant H.K., Whitaker J. (2015). Biochar suppresses N2O emissions while maintaining N availability in a sandy loam soil. Soil Biol. Biochem..

[b0065] Chang T.-G., Zhu X.-G. (2017). Source–sink interaction: a century old concept under the light of modern molecular systems biology. J. Exp. Bot..

[b0070] Changxun G, Zhiyong P, Shu’ang P. 2016. Effect of biochar on the growth of Poncirus trifoliata (L.) Raf. seedlings in Gannan acidic red soil. Soil Science and Plant Nutrition.62:194-200.

[b0075] Chauhan, B.S.; Jabran, K.; Mahajan, G. Rice Production Worldwide; Springer: Berlin/Heidelberg, Germany, 2017.

[b0080] Chen W., Meng J., Han X., Lan Y., Zhang W. (2019). Past, present, and future of biochar. Biochar In..

[b0085] Chen Y., Liu L., Guo Q., Zhu Z., Zhang L. (2016). Effects of different water management options and fertilizer supply on photosynthesis, fluorescence parameters and water use efficiency of Prunella vulgaris seedlings. Biol. Res..

[b0095] DeFries R.S., Ellis E.C., Chapin F.S., Matson P.A., Turner B., Agrawal A., Crutzen P.J., Field C., Gleick P., Kareiva P.M. (2012). Planetary opportunities: a social contract for global change science to contribute to a sustainable future. Bioscience.

[b0100] Elad Y., David D.R., Harel Y.M., Borenshtein M., Kalifa H.B., Silber A., Graber E.R. (2010). Induction of systemic resistance in plants by biochar, a soil-applied carbon sequestering agent. Phytopathology..

[b0105] Enders A., Hanley K., Whitman T., Joseph S., Lehmann J. (2012). Characterization of biochars to evaluate recalcitrance and agronomic performance. Bioresource technology.

[b0110] Ezawa T., Yamamoto K., Yoshida S. (2002). Enhancement of the effectiveness of indigenous arbuscular mycorrhizal fungi by inorganic soil amendments. Soil Science and Plant Nutrition..

[b9000] FAOSTAT, F. (2019). Food and Agriculture Organization of the United Nations-Statistic Division https://www.fao.org/faostat/en/#data.

[b0120] Farhangi-Abriz S., Torabian S. (2018). Biochar improved nodulation and nitrogen metabolism of soybean under salt stress. Symbiosis..

[b0125] Forde BG, Lea PJ. 2007. Glutamate in plants: metabolism, regulation, and signalling. Journal of experimental botany.58:2339-2358. 10.1093/jxb/erm121 17578865

[b0130] Funayama K, Kojima S, Tabuchi-Kobayashi M, Sawa Y, Nakayama Y, Hayakawa T, Yamaya T. 2013. Cytosolic glutamine synthetase1; 2 is responsible for the primary assimilation of ammonium in rice roots. Plant and cell physiology.54:934-943. 10.1093/pcp/pct046 23509111

[b0135] Glass A.D.M. (1995). Nitrogen absorption in higher plants. Nitrogen nutrition in higher plants..

[b0140] Huang D., Liu L., Zeng G., Xu P., Huang C., Deng L., Wang R., Wan J. (2017). The effects of rice straw biochar on indigenous microbial community and enzymes activity in heavy metal-contaminated sediment. Chemosphere.

[b0145] Huang T., Yang H., Huang C., Ju X. (2017). Effect of fertilizer N rates and straw management on yield-scaled nitrous oxide emissions in a maize-wheat double cropping system. Field Crops Research..

[b0150] Huang M., Yang L., Qin H., Jiang L., Zou Y. (2014). Fertilizer nitrogen uptake by rice increased by biochar application. Biol. Fertil. Soils.

[b0155] Iqbal A., He L., Khan A., Wei S., Akhtar K., Ali I., Ullah S., Munsif F., Zhao Q., Jiang L. (2019). Organic manure coupled with inorganic fertilizer: An approach for the sustainable production of rice by improving soil properties and nitrogen use efficiency. Agronomy..

[b0160] Jha SK, Gao Y, Liu H, Huang Z, Wang G, Liang Y, Duan A. 2017. Root development and water uptake in winter wheat under different irrigation methods and scheduling for North China. Agricultural water management.182:139-150.

[b0165] Jones D.L., Rousk J., Edwards-Jones G., DeLuca T.H., Murphy D.V. (2012). Biochar-mediated changes in soil quality and plant growth in a three year field trial. Soil Biol. Biochem..

[b0170] Kichey T., Le Gouis J., Sangwan B., Hirel B., Dubois F. (2005). Changes in the cellular and subcellular localization of glutamine synthetase and glutamate dehydrogenase during flag leaf senescence in wheat (Triticum aestivum L.). Plant Cell Physiol..

[b0175] Krapp A., Berthomé R., Orsel M., Mercey-Boutet S., Yu A., Castaings L., Elftieh S., Major H., Renou J.-P., Daniel- Vedele F. (2011). Arabidopsis roots and shoots show distinct temporal adaptation patterns toward nitrogen starvation. Plant Physiol..

[b0180] Laghari M., Mirjat M.S., Hu Z., Fazal S., Xiao B., Hu M., Chen Z., Guo D. (2015). Effects of biochar application rate on sandy desert soil properties and sorghum growth. Catena..

[b0185] Laird D., Fleming P., Wang B., Horton R., Karlen D. (2010). Biochar impact on nutrient leaching from a Midwestern agricultural soil. Geoderma.

[b0190] Lehmann J., Rillig M.C., Thies J., Masiello C.A., Hockaday W.C., Crowley D. (2011). Biochar effects on soil biota–a review. Soil biology and biochemistry.

[b0195] Li G., Xue L., Gu W., Yang C., Wang S., Ling Q., Qin X., Ding Y. (2009). Comparison of yield components and plant type characteristics of high-yield rice between Taoyuan, a ‘special eco-site’and Nanjing. China. Field Crops Research..

[b0200] Li Y., Li Y., Chang S.X., Yang Y., Fu S., Jiang P., Luo Y., Yang M., Chen Z., Hu S. (2018). Biochar reduces soil heterotrophic respiration in a subtropical plantation through increasing soil organic carbon recalcitrancy and decreasing carbon-degrading microbial activity. Soil Biol. Biochem..

[b0205] Liang F., G-t L.I., Q-m L.I.N., X-r Z.H.A.O. (2014). Crop yield and soil properties in the first 3 years after biochar application to a calcareous soil. J. Integrative Agriculture..

[b0210] Lillo C. (1984). Diurnal variations of nitrite reductase, glutamine synthetase, glutamate synthase, alanine aminotransferase and aspartate aminotransferase in barley leaves. Physiol. Plant..

[b9010] Liu X., Zhang A., Ji C., Joseph S., Bian R., Li L., Paz-Ferreiro J. (2013). Biochar’s effect on crop productivity and the dependence on experimental conditions—a meta-analysis of literature data. Plant and soil.

[b0215] Masclaux-Daubresse C., Reisdorf-Cren M., Pageau K., Lelandais M., Grandjean O., Kronenberger J., Valadier M.-H., Feraud M., Jouglet T., Suzuki A. (2006). Glutamine synthetase-glutamate synthase pathway and glutamate dehydrogenase play distinct roles in the sink-source nitrogen cycle in tobacco. Plant physiology.

[b0220] McBratney A., Field D.J., Koch A. (2014). The dimensions of soil security. Geoderma.

[b0230] Michaud M., Jouhet J. (2019). Lipid trafficking at membrane contact sites during plant development and stress response. Front. Plant Sci..

[b0235] Mu X., Chen Q., Chen F., Yuan L., Mi G. (2016). Within-leaf nitrogen allocation in adaptation to low nitrogen supply in maize during grain-filling stage. Front. Plant Sci..

[b0240] Mukherjee A., Lal R., Zimmerman A. (2014). Effects of biochar and other amendments on the physical properties and greenhouse gas emissions of an artificially degraded soil. Sci. Total Environ..

[b0245] Peng S., Buresh R.J., Huang J., Yang J., Zou Y., Zhong X., Wang G., Zhang F. (2006). Strategies for overcoming low agronomic nitrogen use efficiency in irrigated rice systems in China. Field Crops Research..

[b0250] Porra R., Thompson W., Kriedemann P. (1989). Determination of accurate extinction coefficients and simultaneous equations for assaying chlorophylls a and b extracted with four different solvents: verification of the concentration of chlorophyll standards by atomic absorption spectroscopy. Biochimica et Biophysica Acta (BBA)-Bioenergetics..

[b0255] Pratelli R., Pilot G. (2014). Regulation of amino acid metabolic enzymes and transporters in plants. J. Exp. Bot..

[b0260] Prendergast-Miller M., Duvall M., Sohi S. (2014). Biochar–root interactions are mediated by biochar nutrient content and impacts on soil nutrient availability. Eur. J. Soil Sci..

[b0265] Razaq M., Shen H-l, Sher H., Zhang P. (2017). Influence of biochar and nitrogen on fine root morphology, physiology, and chemistry of Acer mono. Scientific reports.

[b0270] Rennenberg H., Dannenmann M. (2015). Nitrogen nutrition of trees in temperate forests—the significance of nitrogen availability in the pedosphere and atmosphere. Forests..

[b0275] Robin P. 1979. Etude de quelques conditions d'extraction de la nitrate réductase des racines et des feuilles de plantules de maïs.

[b0280] Rondon M.A., Lehmann J., Ramírez J., Hurtado M. (2007). Biological nitrogen fixation by common beans (Phaseolus vulgaris L.) increases with bio-char additions. Biol. Fertil. Soils.

[b0285] Sahrawat K., Burford J. (1982). Modification of the alkaline permanganate method for assessing the availability of soil nitrogen in upland soils. Soil Sci..

[b0290] Sanchez-Bragado R, Molero G, Reynolds MP, Araus JL. 2016. Photosynthetic contribution of the ear to grain filling in wheat: a comparison of different methodologies for evaluation. Journal of experimental botany.67:2787- 2798. 10.1093/jxb/erw116 PMC4861024 27012283

[b0295] Schroeder J.I., Delhaize E., Frommer W.B., Guerinot M.L., Harrison M.J., Herrera-Estrella L., Horie T., Kochian L.V., Munns R., Nishizawa N.K. (2013). Using membrane transporters to improve crops for sustainable food production. Nature.

[b0300] Shu S., Tang Y., Yuan Y., Sun J., Zhong M., Guo S. (2016). The role of 24-epibrassinolide in the regulation of photosynthetic characteristics and nitrogen metabolism of tomato seedlings under a combined low temperature and weak light stress. Plant Physiol. Biochem..

[b0305] Singh R.P., Srivastava H. (1986). Increase in glutamate synthase (NADH) activity in maize seedlings in response to nitrate and ammonium nitrogen. Physiol. Plant..

[b0310] Slavich P., Sinclair K., Morris S., Kimber S., Downie A., Van Zwieten L. (2013). Contrasting effects of manure and green waste biochars on the properties of an acidic ferralsol and productivity of a subtropical pasture. Plant Soil.

[b0315] Speratti A.B., Romanyà J., Garcia-Pausas J., Johnson M.S. (2018). Determining the stability of sugarcane filtercake biochar in soils with contrasting levels of organic matter. Agriculture..

[b0320] Spokas K.A., Koskinen W.C., Baker J.M., Reicosky D.C. (2009). Impacts of woodchip biochar additions on greenhouse gas production and sorption/degradation of two herbicides in a Minnesota soil. Chemosphere.

[b0325] Taghizadeh-Toosi A., Clough T.J., Sherlock R.R., Condron L.M. (2012). Biochar adsorbed ammonia is bioavailable. Plant Soil.

[b0330] Thomas S.C., Gale N. (2015). Biochar and forest restoration: a review and meta-analysis of tree growth responses. New Forest..

[b0335] Tian X., Li C., Zhang M., Wan Y., Xie Z., Chen B., Li W. (2018). Biochar derived from corn straw affected availability and distribution of soil nutrients and cotton yield. PLoS ONE.

[b0340] Ullah S., Liang H., Ali I., Zhao Q., Iqbal A., Wei S., Jiang L. (2020). Biochar coupled with contrasting nitrogen sources mediated changes in carbon and nitrogen pools, microbial and enzymatic activity in paddy soil. J. Saudi Chemical Society.

[b0345] Ullah, S., Ali, I., Liang, H., Zhao, Q., Wei, S., Muhammad, I., ... & Jiang, L. (2021)An approach to sustainable agriculture by untangling the fate of contrasting nitrogen sources in double‐season rice grown with and without biochar. GCB Bioenergy, 13, 382-392.

[b0350] Uribelarrea M., Crafts-Brandner S.J., Below F.E. (2009). Physiological N response of field-grown maize hybrids (Zea mays L.) with divergent yield potential and grain protein concentration. Plant Soil.

[b0355] Vassilev S.V., Baxter D., Andersen L.K., Vassileva C.G. (2013). An overview of the composition and application of biomass ash. Part 1. Phase–mineral and chemical composition and classification. Fuel.

[b0360] Venema J.H., Villerius L., van Hasselt P.R. (2000). Effect of acclimation to suboptimal temperature on chilling-induced photodamage: comparison between a domestic and a high-altitude wild Lycopersicon species. Plant Sci..

[b0365] Wang F., Wang G., Li X., Huang J., Zheng J. (2008). Heredity, physiology and mapping of a chlorophyll content gene of rice (Oryza sativa L.). J. plant physiology.

[b0370] Wang T., Camps-Arbestain M., Hedley M., Bishop P. (2012). Predicting phosphorus bioavailability from high-ash biochars. Plant Soil.

[b0375] Wang, H., Baek, K., Xue, J., Li, Y., & Beiyuan, J. (2020). Preface—Biochar and agricultural sustainability.

[b0380] Woods, W. I., Falcão, N. P., & Teixeira, W. G. (2006). Biochar trials aim to enrich soil for smallholders. Nature, 443(7108), 144-144. 10.1038/443144b 16971925

[b0385] Wu F., Jia Z., Wang S., Chang S.X., Startsev A. (2013). Contrasting effects of wheat straw and its biochar on greenhouse gas emissions and enzyme activities in a Chernozemic soil. Biol. Fertil. Soils.

[b0390] Xi J-c, Kong Q-q, Wang X-g (2015). Spatial polarization of villages in tourist destinations: A case study from Yesanpo China. J. Mountain Sci..

[b0395] Xiang Y., Deng Q., Duan H., Guo Y. (2017). Effects of biochar application on root traits: a meta-analysis. GCB bioenergy.

[b0400] Xu D-Q, Shen Y-K. 2002. Photosynthetic efficiency and crop yield. Handbook of plant and crop physiology Marcel Dekker, New York, NY.821-834.

[b0405] Xu G., Fan X., Miller A.J. (2012). Plant nitrogen assimilation and use efficiency. Annual review of plant biology.

[b0410] Xu X., Li Y., Wang B., Hu J., Liao Y. (2015). Salt stress induced sex-related spatial heterogeneity of gas exchange rates over the leaf surface in Populus cathayana Rehd. Acta physiologiae plantarum..

[b0415] Yanai Y., Toyota K., Okazaki M. (2007). Effects of charcoal addition on N2O emissions from soil resulting from rewetting air-dried soil in short-term laboratory experiments. Soil science and plant nutrition.

[b0420] Yang J., Zhang J. (2010). Grain-filling problem in ‘super’rice. Journal of experimental botany.

[b0425] Yang L., Liao F., Huang M., Yang L., Li Y. (2015). Biochar improves sugarcane seedling root and soil properties under a pot experiment. Sugar tech.

[b0430] Younis U., Athar M., Malik S.A., Raza Shah M.H., Mahmood S. (2015). Biochar impact on physiological and biochemical attributes of Spinach (Spinacia oleracea L.) in nickel contaminated soil. Global J. Environmental Science Management.

[b0435] Yu H., Zou W., Chen J., Chen H., Yu Z., Huang J., Tang H., Wei X., Gao B. (2019). Biochar amendment improves crop production in problem soils: A review. J. environmental management.

[b0440] Yu L, Jiao Y-j, Zhao X-r, Li G-t, Zhao L-x, Meng H-b. 2014. Improvement to maize growth caused by biochars derived from six feedstocks prepared at three different temperatures. Journal of Integrative Agriculture.13:533-540.

[b0445] Zavalloni C., Alberti G., Biasiol S., Delle Vedove G., Fornasier F., Liu J., Peressotti A. (2011). Microbial mineralization of biochar and wheat straw mixture in soil: a short-term study. Appl. Soil Ecol..

[b0450] Zhang K., Fang Z., Liang Y., Tian J. (2009). Genetic dissection of chlorophyll content at different growth stages in common wheat. J. Genetics.

